# Importance of PERK pathway modulation on colorectal cancer management: a systematic review

**DOI:** 10.1186/s12885-025-14952-w

**Published:** 2025-10-03

**Authors:** Marzieh Nemati, Sanaz Dastghaib, Zahra Hosseinzadeh, Mina Molayem, Morvarid Siri, Bahareh Ebrahimi, Zohreh Bagheri

**Affiliations:** 1 Department of Medicine, Indiana University School of Medicine, Indianapolis, IN USA; 2 Endocrinology and Metabolism Research Center, Shiraz University of Medical Sciences, Shiraz, Iran; 3 Autophagy Research Center, Shiraz University of Medical Sciences, Shiraz, Iran; 4 Shiraz Geriatric Research Center, Shiraz University of Medical Sciences, Shiraz, Iran; 5 Department of physiology, Shiraz University of Medical Sciences, Shiraz, Iran

**Keywords:** Colorectal cancer, Endoplasmic reticulum stress, Unfolded protein response, Protein kinase RNA like endoplasmic reticulum kinase

## Abstract

**Background:**

The protein kinase RNA-like endoplasmic reticulum kinase (PERK) branch of the Unfolded Protein Response (UPR) plays a complex and context-dependent role in the colorectal cancer (CRC). While some studies indicate that PERK activation suppresses tumor growth by inducing apoptosis and limiting proliferation, others suggest that it may promote tumor progression by supporting cancer cell survival under stress. This systematic review aims to clarify the dual role of PERK signaling in CRC and evaluate its potential as a therapeutic target.

**Methods:**

We included full-text English-language studies investigating the role of PERK signaling in CRC using in vitro and/or animal models. Studies on non-CRC malignancies or unrelated mechanisms were excluded. Searches were conducted in PubMed, Web of Science (WOS), and Scopus using relevant keywords.

**Results:**

A total of 395 articles were initially identified. After removing duplicates (*n* = 173), review articles (*n* = 11), and unrelated studies (*n* = 66), 45 studies met the inclusion criteria. Most of these (*n* = 36) used in vitro models, with the HCT-116 cell line being the most frequently used (*n* = 19). While most studies (*n* = 36) reported anti-tumorigenic effects associated with PERK activation, several identified conditions under which PERK signaling may support tumor progression. These conflicting findings may be attributed to differences in experimental models, PERK modulation strategies, and endoplasmic reticulum stress induction methods.

**Conclusions:**

This review highlights the dual and context-dependent nature of PERK pathway activation in CRC. Although PERK often appears to exert tumor-suppressive effects, evidence also points to its tumor-promoting potential under certain conditions. A nuanced understanding of these roles is crucial for developing PERK-targeted therapies in CRC.

**Trial registration:**

This systematic review has been registered in PROSPERO (International Prospective Register of Systematic Reviews) with the registration number CRD42023241342.

**Supplementary Information:**

The online version contains supplementary material available at 10.1186/s12885-025-14952-w.

## Background

Colorectal cancer (CRC) is the third most commonly diagnosed cancer and the second leading cause of cancer death worldwide [1]. Surgery is the primary treatment approach, and chemotherapy in combination with radiation therapy is often used for advanced CRC. Despite significant advances in these treatment efforts, the survival rate of patients in advanced stages remains low [2]. One of the most widely studied mechanism of cancer treatment resistance is the activation of the ‘unfolded protein response’ (UPR) as part of a stress-adaptation program [3–5].

The UPR is initiated in response to the accumulation of misfolded or unfolded proteins in the endoplasmic reticulum (ER), a vital organelle where newly synthesized proteins must be folded and assembled before being transported to their destination [6]. Several physiological and pathological conditions can disrupt ER homeostasis, causing ER stress and UPR activation. These conditions include protein synthesis in excess of ER capacity, accumulation of misfolded proteins, inflammation, altered activity of oncogenes and tumor suppressor genes, nutrient deprivation, and hypoxia in normal cells [7–9].

The UPR is mediated by three major ER transmembrane protein sensors: protein kinase RNA-like endoplasmic reticulum kinase (PERK), activating transcription factor 6α (ATF6α), and inositol-requiring enzyme-1α (IRE1α). In homeostasis, the main ER chaperone, glucose-related protein 78 (GRP78), regulates the UPR by binding and inactivating these three sensors. However, during ER stress, high levels of folded membranes lead to dissociation of GRP78 and release of sensors to initiate UPR signaling.

PERK, a UPR signaling partner protein, also promotes ATF4 translation, leading to the expression of the transcription factor C/EBP homologous protein (CHOP) and activation of other downstream signaling molecules that contribute to the recovery of protein translation [10]. PERK can regulate the survival and death of tumor cells depending on the context [11]. For example, activation of the PERK pathway is associated with the development of β-cell insulinoma, while it leads to reduced proliferation in gastric cancer cell lines [12, 13].

Several studies have demonstrated that PERK activation and phosphorylation of its downstream effectors can trigger apoptotic-signaling cascades, leading to tumor-suppressive outcomes [14, 15].

However, other evidence suggests that PERK signaling may facilitate cancer cell survival and progression under chronic stress conditions. This conflicting evidence highlights a critical knowledge gap regarding the precise, context-dependent role of PERK signaling in colorectal cancer (CRC). Specifically, it remains unclear under which conditions PERK acts as a tumor suppressor versus a tumor promoter in CRC models, and how experimental variations (cell lines, stress inducers, PERK modulators) contribute to these divergent outcomes.

## Materials and methods

### Focused question

This systematic review was conducted in accordance with the PRISMA statement and aimed to address the question: “What is the precise role of PERK pathway activation in colorectal cancer (CRC) progression?”

### Search and study selection

A systematic literature search was conducted to identify studies examining the role of PERK in colorectal cancer. The search strategy used the following string: (colorectal cancer[Title/Abstract]) AND (PERK[Title/Abstract]) and was applied across three databases—PubMed, Scopus, and Web of Science—without restrictions on publication date. The search was completed in [insert month and year], and only articles published in English were considered. The initial search yielded 383 records: 139 from PubMed, 111 from Scopus, and 133 from Web of Science. After removing 169 duplicate entries, 214 articles remained for screening. Titles and abstracts were reviewed for relevance, resulting in the exclusion of 8 review articles and 162 unrelated studies. A total of 44 articles were deemed eligible and included for further analysis. The complete search strategy and screening flow are provided in Supplementary File (Appendix.1).

Removal of duplicates was performed independently by three researchers through manual screening. Inclusion criteria included articles published in English with full text available and focusing on colorectal cancer progression and the mechanism of the PERK pathway. Abstracts of articles that were not published in full, review articles, malignancies other than colorectal cancer, and mechanisms other than PERK were excluded from the study. There were no restrictions on the type of CRC cell lines, whether human or animal, or age group. Risk of bias in the included in vivo studies was assessed using the SYRCLE Risk of Bias tool, which evaluates domains such as selection bias, performance bias, detection bias, and reporting bias. The results of this assessment are summarized in Supplementary File (Appendix.2) and were considered when interpreting the strength and reliability of the evidence. Since formal risk of bias tools like SYRCLE is not applicable to cell line experiments, we did not perform a formal bias assessment for these studies. However, we have noted this limitation. Data were extracted based on the type of study (in vitro or in vivo), source of CRC cells, method of PERK modulation (activation or inhibition), approach to assessing PERK signaling, and reported outcomes. For synthesis, studies were grouped by experimental model, PERK modulation strategy, and observed effects on CRC progression. The findings were then thematically analyzed to identify consistent patterns and highlight contradictory results across studies.

The search was repeated in February 2024 to identify any reports that had emerged during the manuscript development period. Our systematic review has been registered in PROSPERO (International Prospective Register of Systematic Reviews) with the registration number CRD42023241342.

## Results

**Fig. 1:**
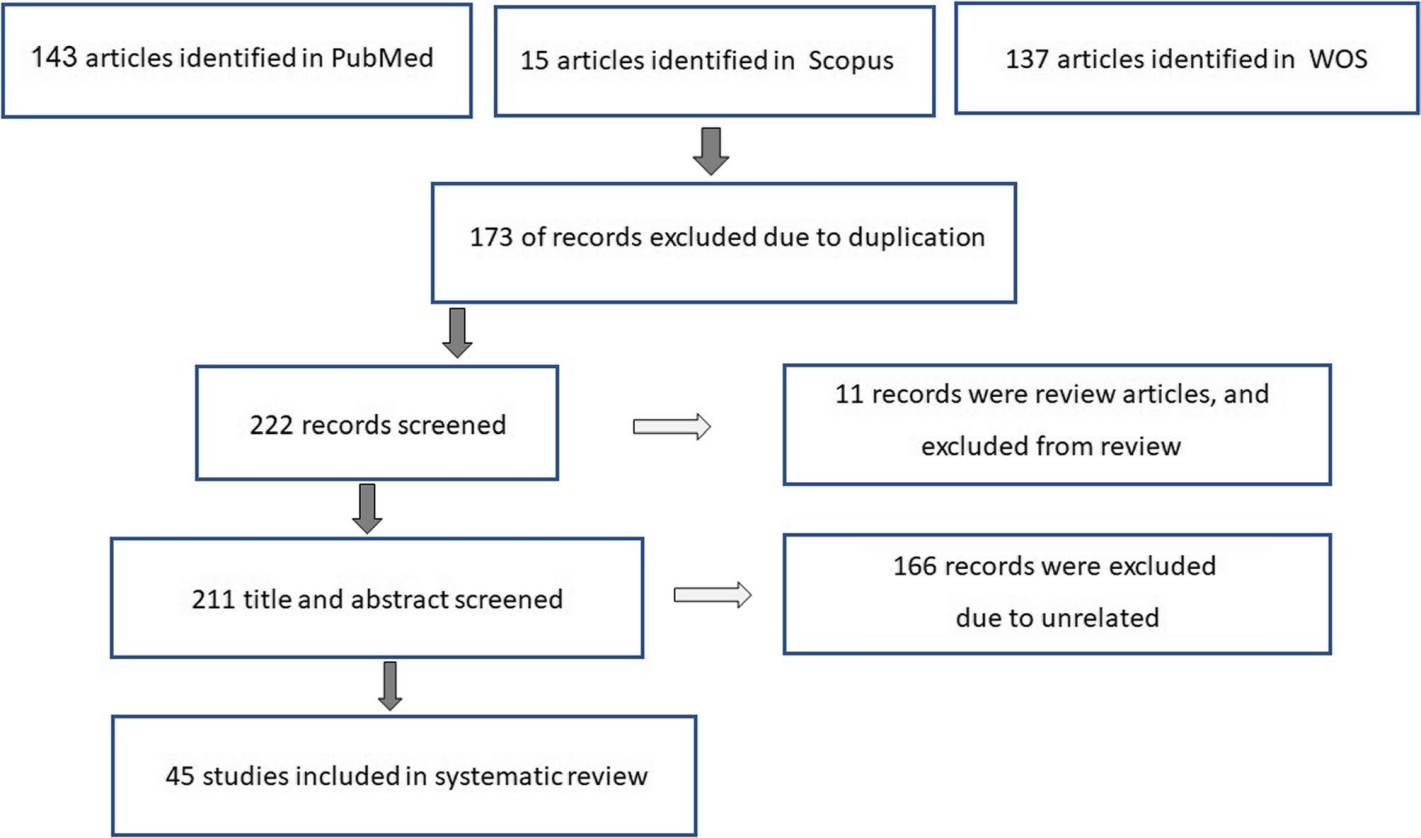
PRISMA Flow Diagram of Study Selection. This figure illustrates the systematic screening process for identifying relevant studies on PERK in colorectal cancer (CRC). It includes the number of records retrieved from each database (PubMed, Scopus, Web of Science), duplicates removed, articles screened, excluded (with reasons), and final studies included. Abbreviations: CRC, colorectal cancer; PERK, protein kinase RNA-like endoplasmic reticulum kinase

Initially, a total of 295 articles (PubMed: 143, Scopus: 15, WOS: 137) were identified. After removing duplicates (173 articles), 122 papers met all inclusion criteria and were selected. In addition, 11 review articles and 66 irrelevant papers were excluded (Fig. 1). Among the remaining original articles, only 45 included results about the role of PERK arm activity in CRC control. Among these articles, thirty-six articles were related to suppressive effects and seven articles were related to tumorigenic effects (Table 1), which will be explained below. A summary table is included to facilitate comparison of key studies and methodologies (Table 2).

**Table 1:** Summary of included studies investigating PERK in CRC

Cell line Type (Human colorectal cancer cells and normal colon cells) Animal Type	Intervention	Intervention Time (Hour (h))	Assessing signaling molecules		Outcome	Mechanism	Author Year
			signaling Molecules	Assessing Method			
SW480, HT-29, HCT116 NCM460	Combination of 5-fluorouracil and withaferin-A	24 h	PERK	RT-PCR Western blotting	The combination of WA and 5-FU decreases cell viability and showed anti-proliferative effect in CRC cells.	Upregulates the expression of PERK	Alnuqaydan, A. M. 2020
HT-29, HCT116, Caco-2	Indomethacin	1 h	eIF2α	Western blotting	Indomethacin was also found to markedly increase apoptosis in HT-29 cells exposed to low-dose cisplatin, suggesting that INDO-triggered inhibition of protein synthesis may sensitize CRC cells to cisplatin treatment.	Causing rapid phosphorylation of eif2α and did not affect the activity of PERK	Brunelli, C. 2012
HCT116, HT-29, Caco-2	Ethanol	3 h and 24 h	PERK	Western blotting	Ethanol-Mediated-Stress Promotes autophagic survival and aggressiveness of colon cancer cells.	Increased the expression of PERK	Cernigliaro, C. 2019
SW620, HT-29	Aloe-emodin (AE)	24 h	PERK	Western blotting	AE suppressed cell viability and induced cell apoptosis in SW620 and HT-29 cell lines.	Upregulation of PERK	Chunsheng Cheng, C. And Dong, D. 2018
MIP-101(Control, resistant to 5FU, overexpressing SPARC), RKO (Control, resistant to 5FU, resistant to CPT-11)	SPARC siRNAs		PERK	Western blotting	The interaction between SPARC and GRP78 induce ER stress-associated cell death during chemotherapy treatment in CRC.	Activation of PERK-eif2α	Chern, Y. 2019
HCT116	6,7-Dihydroxy-2-(4’-hydroxyphenyl)naphthalene	Different concentrations for 24–48 h or with 20 µM PNAP-6 at different time intervals	PERK	Western blotting	PNAP-6 inhibited HCT116 cell proliferation.	Increased the expression of PERK	Chiu, C. F. 2019
HCT116	ICD inducers (Mitoxantrone and Oxaliplatin) and miR-27a_KD and miR-27a_OE	0, 6, 12, and 24 h	PERK	Western blotting	MiR-27a impairs the cell response to drug-induced ICD through the regulatory axis with calreticulin.	Phosphorylation of PERK and its downstream factor eif2a, already high in mir27a_KD cells	Colangelo, T. 2016
LoVo Xenograft models nude male mice	Overexpression/silencing of FAM172A		PERK	Western blotting	FAM172A functioned as a tumor suppressor in colorectal carcinoma.	FAM172A overexpression inhibited the expressions of PERK	Cui, C. 2016
HCT116, DLD-1	Smad7 knockdown (Smad7 antisense or Smad7 sense oligonucleotide/PKR-siRNA or scrambled- siRNA)	24 h	PERK	Western blotting	SMAD7 knockdown promotes CRC cell death.	Activation of PERK	De Simone, V. 2017
HCT116-p53null cells, SW480 Atf3 null/p53 null double knockout (DKO) mice	1) siATF3 2) co-treatment of TNF-related apoptosis-inducing ligand (TRAIL) with ZERO or calcium channel blockers	24 h	PERK ATF3 ATF4	Western blotting	ATF3, which up-regulates DR5 independently of p53 and therefore induces ultimate TRAIL sensitization of cancer cells.	Activation of PERK–eIF2α	Edagawa, M. 2014
HCT116, SW480	NKP-1339 (a clinically investigated ruthenium-based metal complex)	24 h	PERK	Western blotting	Molecular mode of action of NKP-1339 involves ER- and ROS-related effects in colon carcinoma cell lines.	Upregulation of PERK	Flocke, L. S. 2016
HCT116, LoVo	Inhibition of ATF4(shRNA) in CRC cells under the GD condition and in ATF4-overexpression CRC cells	48 h	PEKR ATF4	Western blotting Real-Time qRT-PCR	GD induces the MDR phenotype of CRC cells by activating PEKR/ATF4 signaling.	Activating PEKR/ATF4	Hu, Y. L. 2016
HCT116, SW620, SW1116, HT-29	Thapsigargin, MALAT1 knockdown (siRNA-MALAT1)	24 h	ATF4 eIF2	Real-Time qRT-PCR Western blotting	Thapsigargin promotes colorectal cancer cell migration through upregulation of lncRNA MALAT1.	Activating PEKR signaling	Jiang, X. 2020
HCT116, HT-29, DLD-1 Female BALB/c nude mice	Combination of Cannabidiol and TRAIL	24 h	PERK	Western blotting	Cannabidiol induces significant TRAIL-induced apoptosis of colorectal cancer cells, which is mediated by ER stress.combination of TRAIL and Cannabidiol decreased tumor growth in xenograft models.	Activating PEKR	Kim, J. L. 2019
CX-1	PDT (Photodynamic therapy)- Sinoporphyrin sodium (DVDMS)	4 h	PERK	Western blotting Real-Time qRT-PCR	MiR-7112-3p directly targeted PERK and further regulated PERK/ATF4/CHOP/caspase cascade pathway, resulting in enhanced apoptosis in CX-1 cells treated with DVDMS-PDT.	Activating PEKR	Kong, F. H. 2020
SNU-C5	Non-thermal gas plasma (NTGP)	24 h	PERK	Western blotting	Plasma-generated ROS induce apoptosis in SNU-C5 cells.	Increased the levels of PERK	Kumara, Mhsr 2016
HCT116	Treated with hypoxia (1% O_2_)	0, 4, 8, 16, 24 h	eIF2α	Western blotting	Hypoxia induces autophagy through translational up-regulation of lysosomal proteins in HCT116 cells.	Activation of PERK signaling pathway	Lai, M. C. 2016
HCT116, SW480, HT-29	TRAIL	1–4 h	PERK	Western blotting	TRAIL induces caspase-mediated ER stress which facilitates apoptotic death through the activation of the PERK-eIF2a-ATF4-CHOP signal transduction pathways.	Activation of the PERK	Lee, D. H. 2016
SW480, COLO 205 Xenograft mouse model (male BALB/c nude mice)	Knockdown of CEMIP (CEMIP shRNA)		PERK	Western blotting	Knockdown of CEMIP suppresses proliferation and induces apoptosis in colorectal cancer cells.	Decreased the expression of PERK	Liang, G. 2018
HCT116, HT-29 Xenograft mouse model (nude mice)	Cinobufagin (CBG)	12 h	PERK	Western blotting	CBG-induced CRC cell death is associated with ER stress activation.	Activation of the PERK	Lu, X. S. 2017
HCT116	TRAP1-myc expression vector		PERK	Western blotting	TRAP1 silencing sensitizes cells to apoptosis induced by novel antitumoral drugs that inhibit cap-dependent translation, such as ribavirin or 4EGI-1, and reduces the ability of cells to migrate through the pores of transwell filters.	Activation of the PERK	Matassa, D. S. 2013
HT-29	Green tea epigallocatechin-3-gallate (EGCG)	24, 48, 72 h	PERK	Western blotting	EGCG has the potential to inhibit colorectal cancer cells through the induction of ER stress.	Upregulation of PERK	Nesran, Z. N. 2019
Caco-2, HCT116	RHBDD2 overexpression and silencing + 5-FU	72 h	PERK	Western blotting, Real-Time qRT-PCR	RHBDD2 overexpression confers colon cancer cells resistance to 5Fu treatment.	Downregulating PERK	Palma, S. 2020
HCT-8, HCT-8/5-FU, DLD-1 Xenograft models (Male nude mice)	Ciclopirox (CPX)	cell:48 h mice:12 days	PERK	Western blotting	CPX through the disruption of cellular bioenergetics and activating PERK dependent ER stress to drive cell death and overcome drug resistance in CRC.	Increased PERK expression	Qi, J. 2020
HT-29 Immuno-competent BALB/c mice	Curcumin + Sildenafil	cell: 24 h mice: 5 days	PERK	Immuno-Fluorescence	Curcumin interacts with sildenafil to kill GI tumor cells via endoplasmic reticulum stress and reactive oxygen/nitrogen species.	Increased PERK	Rossi, A. 2017
CT26	Nanosecond Pulsed Electric Field (nsPEF)	4 to 24 h	PERK eIF2α	Western blotting, Real-Time qRT-PCR	Nspef activate PERK, and induce ER stress accompanied by ICD (immunogenic cell death).	Activation of the PERK	Rossi, A. 2019
HT-29	GSK2606414 and Thapsigargin	3 h	eIF2α p-eIF2α	Western blotting	GSK2606414 inhibitor can significantly inhibit the PERK-dependent signaling pathway.	Inhibit PERK	Rozpedek, W. 2017
HT-29, CCD 841 CoN	42,215 PERK inhibitor	-	eIF2α	Western blotting	42,215 PERK inhibitor is selective only toward cancer cells, since inhibited their viability in a dose- and time-dependent manner and induced their apoptosis and G2/M cell cycle arrest. Furthermore, 42,215 PERK inhibitor evoked significant inhibition of eIF2α phosphorylation.	Inhibit PERK	Rozpedek, W. 2020
HCT116	3’,5-dihydroxy-3,4’,7-trimethoxyflavone (DTMF), A novel semi-synthetic derivative of quercetin	24 h	PERK	Western blotting	DTMF stimulates ROS-mediated oxidative stress, which in turn induces PERK-CHOP and JNK pathway of apoptosis to promote HCT116 cell death.	Induce PERK	Singh, M. P. 2017
SW480, SW620	Docosahexaenoic acid (DHA) + TRAIL	24 h with DHA and 4 h with TRAIL	PERK	Western blotting	DHA supports antiproliferative and apoptotic effects of clinically useful cytokine TRAIL in cancer but not normal human colon cells.	Decrease PERK	Skender, B. 2014
LS174T, SW480, DLD-1	Harbor doxycycline inducible expression of the active forms of either XBP1(s) or ATF61-373		PERK	Western blotting, Real-Time Qrt-Pcr	Expression of UPR effector proteins ATF6 and XBP1 reduce colorectal cancer cell proliferation and stemness by activating PERK signaling.	Activation of PERK	Spaan, C. N. 2019
HCT116, HT-29, CT26 CT26-derived xenograft mouse	2-methoxy-5-amino-N-hydroxybenzamide (herein termed 2–14), Mesalamine (5-ASA)	8 h	PERK	Western blotting	The Mesalamine derivative 2–14 inhibited CRC cell proliferation in vitro and prevented CRC progression in mouse models.	Enhance the phosphorylation of PERK	Stolfi, C. 2010
C57BL/6 mice	Resistant Starch	120 days	eIF2α ATF4	Western blotting, Recombinant Glutathione S-Transferase	Resistant starch in the diet may prevent carcinogenesis of colon epithelial cells, mediated by enhancing apoptosis through an endoplasmic reticulum stress-mediated mitochondrial apoptosis pathway.	Resistant starch diet increased the expression levels of eif2α, ATF-4	Wang, Q. Y. 2018
HCT116, SW620 NCM460 Female SCID mice	Ethyl acetate extract of Cichorium (EAEC)	mice:7days	PERK	Western blotting Real-Time qRT-PCR	Treatment with EAEC-PDT reduced xenograft tumor size. Further evaluation suggested that activation of the PERK pathway mediates these effects, as the apoptotic rate and autophagy flux increased markedly after EAEC-PDT.	Activation of the PERK pathway	Wen, Y. 2019
HCT-15, HCT116, HT-29	ruthenium anticancer drug (KP1339/IT-139)	24 h	PERK	Western blotting	Induces an immunogenic cell death signature in colorectal spheroids in vitro.	Phosphorylation of PERK and eif2a	Wernitznig, D. 2019
COLO 205, HCT-15, LoVo, HT‐29	Cobalt protoporphyrin (CoPP), an inducer of heme oxygenase (HO)−1 and Carbon monoxide-releasing molecules (CORMs)	12 h	PERK	Western blotting	Increased HO-1 protein preferentially induced apoptosis related to higher amount of endogenous ROS levels in poor/malignant human CRC. A pro‐apoptotic role of HO‐1 against the viability of human CRC cells via induction of CO and ER stress.	Induced the phosphorylation of the PERK	Wu, M. S. 2019
HCT-15, COLO 205, HT-20, LoVo	c-Jun N-terminal kinase (JNK) inhibitors SP600125 (SP) and JNK inhibitor V (JNKI)	24 h	PERK	Western blotting	TAX and NOC are able to activate JNK and PERK, which in turn promote the apoptosis and G2/M arrest machinery, including mitochondrial apoptotic cascades and phosphorylation of Cdc25C leading to anti-CRC actions.	Increased the expression of phosphorylated PERK	Wu, M. S. 2020
HCT116, HT-29, SW620, DLD-1	miR-451a expression or silencing BAP31	48 h	PERK	Western blotting	MiR-451a can inhibit proliferation and increase apoptosis through inducing ERS by binding to the 5’-UTR of BAP31 in CRC.	Increased the expression of phosphorylated PERK	Xu, K. 2019
HCT116, HT-29, SW480, LoVo, Caco-2, A549, PC-3, AsPC-1 Apc (Min/+) mice	Tolfenamic acid (TA)	mice:3days cell: 24 h	PERK	Western blotting	ER stress is involved in tolfenamic acid-induced inhibition of colorectal cancer cell growth, which could contribute to antitumorigenesis in a mouse model.	Activation of PERK	Zhang, X. 2013
HCT116, SW480, HT-29, LoVo, Caco-2	3,3’-diindolylmethane(DIM)	24 h	PERK ATF4 ATF3	Western blotting	DIM downregulates cyclin D1 through triggering ER stress in human colorectal cancer cells.	Increased ATF3 and ATF4 expression	Zhang, Y. 2017
HCT116, HT-29 S1Pr2−/− mice	Sphingosine-1-phosphate (S1P)	mice: 5 weeks cell: 24 h	PERK	Western blotting	S1P-induced S1PR2 internalization blunts 5-FU therapy by elevating autophagy-related uracil generation. Strategies for blocking S1PR2 internalization may be effective in sensitizing 5- FU-based chemotherapies.	Activate PERK-elf2α-ATF4 signaling	Zhang, Y. 2020
Male C57BL/6 mice	Naringin, Dextran sodium sulfate (DSS), Azoxymethane (AOM)		PERK	Western blotting	Naringin prevented colitis and colorectal carcinogenesis through suppressing robust ER stressinduced autophagy in colorectal mucosal cells.	Activated PERK phosphorylated eif-2a	Zhang, Y. 2018
HT-29, SW480 Nude mouse	Hypoxia, shGDF15 or negative control shRNA	hypoxia for 12 h	eIF2α	Western blotting	ER stress is dramatically induced by hypoxia exposure and subsequently activated PERK-eIF2α signaling promotes the metastasis via regulating GDF15 expression in CRC cells.	Activated PERK-eif2α signaling	Zhang, H. 2020

This table presents key characteristics of the 44 included studies, including author, year, study type (in vitro/in vivo), model system, PERK-related intervention, and main findings. Studies are grouped by experimental design

Abbreviations: *CRC* colorectal cancer; *PERK* protein kinase RNA-like endoplasmic reticulum kinase; *UPR* unfolded protein response

**Table 2:** A structured summary of key studies categorized by experimental models, outcome measures, and relevance to CRC progression

Study	Experimental Model	Methodology	Main Findings	Limitations
Cheng et al., 2018 (16)	In vitro (SW620, HT-29)	Aloe-emodin treatment	PERK activation induced apoptosis in CRC cells	Lack of in vivo validation
Kim et al., 2019 (22)	In vitro (HCT116, HT-29, DLD-1), In vivo (BALB/c nude mice)	TRAIL + Cannabidiol	Combination therapy enhanced PERK-mediated apoptosis	Requires clinical studies
Liang et al., 2018 (57)	In vitro (SW480, COLO 205), In vivo (BALB/c nude mice)	Knockdown of CEMIP	Suppressed proliferation via PERK downregulation	Focused on a single regulatory pathway
Lu et al., 2017 (35)	In vitro (HCT116, HT-29), In vivo (Nude mice)	Cinobufagin treatment	Induced apoptosis through ER stress and PERK activation	No clinical relevance yet
Zhang et al., 2020 (54)	In vitro (HT-29, SW480), In vivo (S1Pr2−/− mice)	Sphingosine-1-phosphate exposure	PERK activation blunted 5-FU therapy efficacy	Requires translational validation

This table presents a categorized overview of the 44 included studies investigating the role of protein kinase RNA-like endoplasmic reticulum kinase (PERK) in colorectal cancer (CRC). Studies are grouped by experimental model type (e.g., in vitro, in vivo), and each entry includes the primary outcome measures assessed (e.g., cell viability, apoptosis, tumor growth), along with a brief summary of the study’s relevance to CRC progression. The table highlights mechanistic insights, therapeutic implications, and consistency of findings across models

Abbreviations: *CRC* colorectal cancer; *PERK* protein kinase RNA-like endoplasmic reticulum kinase; *UPR* unfolded protein response

## Role of PERK on CRC

The PERK pathway functions as an antitumor or tumor-progressive regulator in CRC under different conditions. This regulatory function can be induced through activation and suppression of the PERK arm. Most studies show that the activation of the PERK branch leads to the induction of anti-tumorigenic effects in CRC. The following section discusses these studies based on the examination of antitumorigenic or tumorigenic effects. Additionally, it examines how modulation of PERK arm activity affects CRC progression.

### PERK as an anti-tumorigenic regulator

#### Natural and chemical compounds

Several studies have shown that various natural compounds, including Aloe-emodin (AE) (derived from Aloe Vera) [16], the polyphenolic compound found in the green tea (EGCG) [17], and 3’,5-dihydroxy-3,4’,7-trimethoxyflavone (DTMF) [18], induce cell apoptosis in HT-29, SW-620, and HCT-116 cells through activation of the PERK axis. Combined treatment of sildenafil and curcumin activates the PERK signaling pathway and subsequently causes toxicity and reduced viability in HCT-116 [19]. Treatment of HCT-116 with 3, 3’-diindolylmethane (DIM), a compound enriched in cruciferous vegetables, upregulated PERK pathway protein such as eIF2α, and prompted therapeutic effects [20].

Treatment with bioactive compounds such as withaferin-A (WA) and 5-fluorouracil (5-FU) or acetate extract of cichorium (EAEC) in HCT-116, SW-480, and HT-29 cells has shown its anti-cancer effects by activating the PERK arm, leading to apoptosis and autophagy [16, 21]. The combination of TNF-related apoptosis-inducing ligand (TRAIL), an anticancer agent, with cannabidiol significantly triggered apoptosis and increased cell death in HCT-116, HT29, and DLD-1 cells. Cannabidiol activated death receptor 5 (DR5), ER stress, UPR, and PERK pathway, and subsequently sensitized CRC cell lines to TRAIL and induced apoptosis [22]. Bixin, an apocarotenoid from Bixa orellana seeds, inhibited the proliferation and motility of CaCO2 and SW480 cells. Additionally, it sensitized cells to TRAIL-induced apoptosis. These effects are mediated by activation of the AMPK/PERK/eIF-2α signaling pathway [23]. Furthermore, TRAIL alone increased PERK pathway activity and led to cell death in HCT-116 cells [24].

Naringin, a flavonoid, exerts antitumor effects in CRC by activating UPR sensors such as PERK/eIF-2a signaling and inhibiting ERS-derived induction of autophagy [25]. Furthermore, glucose deprivation activates the PERK pathway in HCT116 and LoVo cells, leading to cell death and resensitization to the drug [26]. Treatment with Shikonin, a naphthoquinone derivative, induced apoptosis and cytotoxicity in the 5-fluorouracil-resistant colorectal cancer cell line SNU-C5/5-FUR. One of the key mechanisms underlying its effects is the activation of PERK signaling [27]. Furthermore, results from two in vivo studies have shown that administration of resistant starch or EAEC to a mouse model of CRC resulted in apoptosis of CRC cells and a reduction in tumor size in the studied animals. These observed effects are attributed, in part, to increased activity of the PERK pathway [21, 28].

In addition to natural compounds, several chemical compounds have also shown antitumor effects in CRC. For example, supplementation of HCT-116, SW-480, HCT-15, LOVO, COLO205, and HT-25 cells with secreted protein, acidic, and cysteine-rich (SPARC), 7-dihydroxy-2-(4′-hydroxyphenyl) naphthalene (PNAP-6), zerumbone (ZER), and celecoxib (CCB), as well as carbon monoxide donor (CORM) and Cobalt protoporphyrin (CoPP), taxol, nocodazole, or tolfenamic acid, resulted in apoptosis and cell death [14, 29–33]. Increased PERK axis activity is responsible for these effects. 6-Cyclohexyl-1-hydroxy-4-methyl-2 (1 H)-pyridinone (CPX) has shown anticancer activity in various cancers. For example, activation of the PERK-eIF2α-ATF4 pathway in both chemoresistant and chemosensitive CRC cell lines leads to apoptosis and cell death in vitro [34]. Application of Cinobufagin (CBG) or 2-methoxy-5-amino-N-hydroxybenzamide upregulated PERK arm activity and induced antiproliferative effects in HT-29 and HCT-116 cells [35, 36]. Furthermore, treatment with NKP-1339 in HCT-116, HCT-15, HT-29, and SW-480 cells induced immunogenic cell death (ICD) and exerted anticancer effects [37, 38]. Docosahexaenoic acid (DHA), an omega-3 fatty acid, increased TRAIL-induced cell death in SW620 cells, and was associated with increased activation of PERK arm that is implicated in inducing these observed effects [39].

The PERK activator, CCT020312 (CCT), reduced CRC cell proliferation in a dose- and time-dependent manner. It also improved the chemosensitivity of drug-sensitive and drug-resistant cells to taxol treatment and significantly reduced their survival rate [40]. NK-1R antagonists, SR140333 and aprepitant, induce cell death in CRC cells. Furthermore, they enhance the efficacy of chemotherapy by increasing sensitivity and hammer resistance to 5-fluorouracil in CRC cells, in part through activation of PERK [41].

Four in vivo studies showed that administration of CBG or 2-methoxy-5-amino-N-hydroxybenzamide to nude mice increased PERK arm activity, inhibited CRC progression, and ultimately reduced tumor size in animal models of CRC [35, 36]. Furthermore, the antitumor activity of CPX was induced by this mechanism in vivo and resulted in suppression of CRC xenograft growth in mice [34]. The combination of CCT and taxol increased PERK activation and resulted in a significant reduction in tumor growth in CRC xenografts [40]. In contrast to these studies, two other research findings showed that the inhibition of PERK arm activity and eIF2α phosphorylation by GSK2606414 or 42215 (PERK inhibitors) may switch the pro-adaptive UPR responses to its pro-apoptotic responses in HT29 cells. This inhibition also leads to cell cycle arrest in CRC cells, such as HT-29 cells [42, 43].

#### Epigenetic factors

Downregulation of miR-7113-3P expression (which is upregulated in CRC) by photodynamic therapy (PDT) in the presence of sinoporphyrin sodium (DVDMS) as a photosensitizer increased PERK arm activity and induced apoptosis in CX-2 cells [44]. Conversely, upregulation of miR-451a (a tumor suppressor) activated the PERK signaling pathway in HCR-116 and SW-620 cells, inhibited cell proliferation, and stimulated apoptosis [45]. Knockdown of Smad7, which is upregulated in CRC, promoted serine-threonine protein kinase RNA (PKR), a regulator of eIF2α. This subsequently increased eIF2α phosphorylation and ATF4/CHOP activation, ultimately causing cell death in CRC [46]. Another study showed that the active form of XBP1 or ATF6, signaling molecules of the other two branches of the UPR, upregulated PERK activity and induced apoptosis in CRC cells [47].

In vivo studies revealed that overexpression of miR-451a in CRC xenograft mice increased PERK activity. This increase led to cell apoptosis, inhibition of CRC cell proliferation, and ultimately inhibition of tumor progression in these animals [45]. In contrast to studies showing antitumor effects by increasing PERK branch activity through various factors, FAM172A, a novel protein, plays a tumor suppressor role. This protein achieves this goal by downregulating PERK expression in tissue samples from CRC patients as well as in LOVO cells [48].

#### Technological methods

Nonthermal gas plasma (NTGP) has recently emerged as a potential application in cancer therapy. Exposure of SNUC5 cells (a type of human colon carcinoma cell) to NTGP induced the expression of UPR proteins such as GRP78 and PERK and subsequently induced cell death in this cell line [49]. Similarly, nanosecond pulsed electric fields (nsPEF), a novel technology for tumor eradication, induced endoplasmic reticulum (ER) stress and immunogenic cell death (ICD) through PERK activation in CT-26 CRC cells [50].

## PERK as a tumor progressive regulator

Some evidence suggests that PERK activation can limit tumor progression, while other evidence emphasizes a positive role for PERK in the development and/or progression of malignancy.

### Natural and chemical compounds

Exposure of HCT-116 cells to hypoxia partially activates the PERK arm, inhibits translation, and induce cell autophagy as an adaptive response to tumor progression [51]. Furthermore, the results of a study showed that under hypoxic conditions, the activity of the PERK-eIF2α branch of the UPR pathway and the expression of growth differentiation factor 15 (GDF15) are increased in HT29 and SW480 cells. GDF15 is known to be a marker of increased metastasis [52].

Long-term ethanol treatment of HCT116, HT29, and Caco-2 cells resulted in increased levels of PERK, ATF6, and CHOP proteins, as well as proteins associated related to the autophagy-signaling pathway. Consequently, these changes led to a survival response in CRC cells [53]. Treatment of HCT-116 cells with Sphingosine-1-phosphate (S1P) promoted internalization of S1P receptors (S1PRs), which in turn stimulated the PERK/eIF2α/ATF4 signaling pathway. This led to the impediment of 5-FU uptake and suppression of its therapeutic effect, ultimately causing CRC progression. Furthermore, S1P administration increased S1PR2 internalization and impaired the treatment efficacy of 5-FU in WT mice compared with S1PR2-/- mice [54].

### Other molecules

Expression of Lung adenocarcinoma metastasis-associated long noncoding RNA transcript 1 (MALAT1) was increased in CRC and subsequently stimulated CRC cell migration through activation of the PERK pathway [55]. Furthermore, high expression of miR-27a, an oncomiRNA, inhibited mitoxantrone-induced immunogenic cell death (ICD) and apoptosis by inactivating the PERK/eIF2α pathway [56]. In addition, two other studies showed that hyaluronan-binding protein (CEMIP) and TNF receptor-associated protein 1 (TRAP1), which promote cell migration, exacerbate CRC progression and metastasis in HCT-116 cells through upregulation of the PERK pathway [57, 58].

## Discussion

CRC is the third most commonly diagnosed cancer type and the second leading cause of cancer-related mortality worldwide [59]. Current treatment approaches for CRC include surgery, radiation therapy, chemotherapy, and combinations thereof. Despite these interventions, none have achieved complete prevention of cancer progression, and survival rates for patients with advanced stages remain low [2].

Understanding the molecular mechanisms driving CRC progression and is a promising approach in combating this malignancy. The role of the UPR, particularly the PERK arm, in cancer progression remains controversial across different cancer types. While some evidence suggests that PERK activation can inhibit tumor progression, other studies have shown its potential to promote cell proliferation. Although the exact factors contributing to this debate remain unknown, it appears that the dual function of PERK in cancer may be influenced by various elements, including the cancer cell type, tumor microenvironment, cancer stage, the models and methods used in the studies [60]. Various in vitro and in vivo studies have re shown vealed that modulation of PERK branch activity produces tumor suppressive or tumor progressive effects in CRC.

Affecting the PERK pathway has shown tumor-suppressive effects. For example, treatment with various agents induces endoplasmic reticulum stress (ERS), subsequently stimulating the UPR and activating PERK. This activation leads to apoptosis, autophagy, reduced cell proliferation and survival, and ultimately contributes to the suppression of CRC [17, 20, 22, 24, 26, 28, 30–32, 50, 61–64]. Yu-Sheng et al. demonstrated that PERK activation contributes to colorectal adenoma reduction. Conversely, another study suggested that ER-induced suppression of autophagy is an alternative mechanism responsible for tumor suppression [25]. Several studies have shown the importance of PERK pathway activity and caspase activity in exerting antitumor effects. For example, the anti-CRC effects of compounds such as EGCG, TRAIL, DHA plus TRAIL, and nsPEF treatment have been attributed in part to caspase activity, particularly caspase 3/7 [17, 24, 39, 50]. Furthermore, activation of the ERS-associated kinase (JNK) pathway [32, 33, 65], and cell cycle arrest in the G0/G1 phase [36] or in G2/M [33] promote apoptosis in CRC.

Ching-Feng and colleagues reported that PNAP increased the expression of p53, p21, and p27, leading to cell cycle arrest and inhibition of CRC cell proliferation [30]. Another study showed a decrease in the expression of p53, a tumor suppressor gene [28]. Treatment with DTMF or application of NTGP resulted in increased ROS production, which led to ERS, elevated mitochondrial calcium levels, and increased calcium release from the endoplasmic reticulum. This cascade of events activated the UPR and the PERK pathway, ultimately inhibiting tumor growth [49, 65]. Matassa and colleagues showed that TRAP1 activates the PERK arm of the pathway, increases phosphorylation of eIf2α, and enhances the synthesis of ATF4 and its downstream targets, BiP/Grp78. This process confers protection against ERS and exerts anti-tumor effects [58]. In contrast to studies suggesting that PERK activation exerts anti-tumor effects, treatment of CRC cells with FAM172A reduced PERK expression and eIf2α phosphorylation, leading to growth inhibition and tumor suppression [48]. Inhibition of PERK by chemical agents, both in vitro and in vivo, shifted the cellular response from pro-adaptive to pro-apoptotic [42, 43].

Modulation of the PERK arm can also have tumorigenic effects. Cernigliaro et al. demonstrated that long-term treatment of CRC cells with ethanol increased ROS production, leading to ERS and activation of the PERK pathway. This, in turn, stimulated the autophagy response and ultimately increased the survival of CRC cells. The use of antioxidants significantly inhibited the autophagy response, suggesting that oxidative stress-induced autophagy promotes cell survival and contributes to tumor progression. Furthermore, ethanol activated the Nrf2/Ho-1 axis and increased MMP-2 and VEGF levels, further promoting CRC progression and increasing CRC cell survival [53]. Incubation of CRC cells with S1P caused internalization of plasma membrane S1PR2 into the ER and increased ER calcium levels. This activates the PERK signaling pathway and subsequently leds to the activation of ATF4, which increases autophagy-signaling molecules. This process disrupts the uptake of exogenous 5-FU, thereby reducing its efficacy against CRC and ultimately increasing tumor progression [54].

Hypoxia activates the UPR and the PERK arm signaling pathway, which in turn regulates the GDF15 expression and cell survival, ultimately promoting tumorigenic effects [52]. During hypoxia, ERS is induced, leading to activation of the UPR and subsequent activation of PERK. PERK regulates translation by phosphorylating eIF2α, thereby increasing ATF4 expression. This leads to activation of LC3-dependent autophagy, which contributes to cell survival under hypoxic conditions [51]. Induction of ERS in CRC cells increases PERK branch activity and subsequently increases the expression of metastasis-associated long non-coding RNA (lncRNA MALAT1), ultimately promoting CRC progression [55]. Furthermore, upregulation of the PERK pathway by hyaluronan-binding protein (CEMIP) promotes cell migration and accelerates CRC progression [57].

The dual role of PERK in CRC progression presents both opportunities and challenges for clinical translation. On one hand, targeting PERK signaling could be a viable therapeutic strategy to induce tumor cell apoptosis or sensitize tumors to chemotherapeutic agents. On the other hand, the tumor-promoting effects of PERK activation under certain conditions such as oxidative stress, hypoxia, or chemoresistance highlight the complexity of its modulation in clinical settings. These contrasting outcomes underscore the need for personalized approaches when designing PERK-targeted therapies. Potential therapeutic applications might include combining PERK inhibitors with agents that suppress autophagy or using PERK activators selectively in tumor types or stages where its anti-tumor effects are dominant. However, clinical translation will require comprehensive preclinical validation, especially in physiologically relevant models and ultimately, well-designed clinical trials to assess efficacy and safety.

This review has several methodological limitations worth noting. First, we did not perform a formal risk-of-bias assessment of the included in vitro studies, which may limit the ability to evaluate the methodological rigor and reliability of the evidence. We acknowledge this limitation to ensure transparency. Second, there is a possibility of publication bias, as studies with positive or significant findings are more likely to be published, potentially skewing the overall interpretation of the PERK pathway’s role in CRC. Third, substantial heterogeneity exists among the included studies in terms of experimental models (in vitro vs. in vivo), cell types, treatment regimens, outcome measures, and CRC subtypes. These variations complicate direct comparisons and may contribute to the conflicting results observed across studies. Taken together, these limitations highlight the need for future research using standardized, well-controlled methodologies to better clarify the therapeutic potential of targeting PERK in CRC.

In conclusion, this systematic review underscores the complex and context-dependent role of PERK pathway modulation in colorectal cancer therapy. PERK signaling can drive both pro-survival and pro-apoptotic responses, depending on cellular conditions. Notably, PERK activation has demonstrated predominantly anti-tumorigenic effects in preclinical models, though its dual nature highlights the importance of tailored therapeutic strategies.

The emergence of PERK-targeted therapies presents a promising approach for CRC treatment. However, to translate these findings into clinical applications, further studies are needed. Future research should incorporate gene silencing and overexpression techniques, such as siRNA or CRISPR/Cas9, alongside well-designed in vivo CRC models to clarify causal relationships and therapeutic mechanisms. Moreover, early-phase clinical trials testing PERK inhibitors or activators—either alone or in combination with chemotherapy or immunotherapy—are warranted to evaluate their therapeutic potential and safety in CRC patients.

## Supplementary Information

Supplementary material 1.

Supplementary material 2.

## Data Availability

No datasets were generated or analysed during the current study.

## Abbreviations

PERK: Protein kinase RNA-like endoplasmic reticulum kinase
UPR: Unfolded Protein Response
ATF6α: Activating Transcription Factor 6α
IRE1α: Inositol-requiring Enzyme-1α
GRP78: Glucose-related Protein 78
CHOP: C/EBP Homologous Protein

## References

[1] Hamanaka RB, Bennett BS, Cullinan SB, Diehl JA. PERK and GCN2 contribute to eIF2α phosphorylation and cell cycle arrest after activation of the unfolded protein response pathway. Mol Biol Cell. 2005;16(12):5493–501. 16176978 10.1091/mbc.E05-03-0268 PMC1289396

[2] Shen T, Huang S. Repositioning the old fungicide ciclopirox for new medical uses. Curr Pharm Des. 2016;22(28):4443–50. 27238364 10.2174/1381612822666160530151209 PMC6623967

[3] Epple LM, Dodd RD, Merz AL, Dechkovskaia AM, Herring M, Winston BA, et al. Induction of the unfolded protein response drives enhanced metabolism and chemoresistance in glioma cells. PLoS One. 2013;8(8):e73267. 24039668 10.1371/journal.pone.0073267 PMC3748289

[4] Lee E, Nichols P, Spicer D, Groshen S, Yu MC, Lee AS. GRP78 as a novel predictor of responsiveness to chemotherapy in breast cancer. Cancer Res. 2006;66(16):7849–53. 16912156 10.1158/0008-5472.CAN-06-1660

[5] Al-Rawashdeh FY, Scriven P, Cameron IC, Vergani PV, Wyld L. Unfolded protein response activation contributes to chemoresistance in hepatocellular carcinoma. Eur J Gastroenterol Hepatol. 2010;22(9):1099–105. 20177386 10.1097/MEG.0b013e3283378405

[6] Walter P, Ron D. The unfolded protein response: from stress pathway to homeostatic regulation. Science. 2011;334(6059):1081–6. 22116877 10.1126/science.1209038

[7] Almanza A, Carlesso A, Chintha C, Creedican S, Doultsinos D, Leuzzi B, et al. Endoplasmic reticulum stress signalling - from basic mechanisms to clinical applications. FEBS J. 2019;286(2):241–78. 30027602 10.1111/febs.14608 PMC7379631

[8] Lin Y, Jiang M, Chen W, Zhao T, Wei Y. Cancer and ER stress: mutual crosstalk between autophagy, oxidative stress and inflammatory response. Biomed Pharmacother. 2019;118:109249. 31351428 10.1016/j.biopha.2019.109249

[9] Nam SM, Jeon YJ. Proteostasis in the endoplasmic reticulum: road to cure. Cancers (Basel). 2019. 10.3390/cancers11111793. 31739582 10.3390/cancers11111793 PMC6895847

[10] Marciniak SJ, Yun CY, Oyadomari S, Novoa I, Zhang Y, Jungreis R, et al. CHOP induces death by promoting protein synthesis and oxidation in the stressed endoplasmic reticulum. Genes Dev. 2004;18(24):3066–77. 15601821 10.1101/gad.1250704 PMC535917

[11] Parmar VM, Schröder M. Sensing Endoplasmic reticulum stress. Adv Exp Med Biol. 2012;738:153–68. 22399379 10.1007/978-1-4614-1680-7_10

[12] Gupta S, McGrath B, Cavener DR. PERK regulates the proliferation and development of insulin-secreting beta-cell tumors in the endocrine pancreas of mice. PLoS One. 2009;4(11):e8008. 19956728 10.1371/journal.pone.0008008 PMC2776514

[13] Seo J, Lee SH, Park SY, Jeong MH, Lee SY, Kim MJ, et al. GPR177 promotes gastric cancer proliferation by suppressing endoplasmic reticulum stress-induced cell death. J Cell Biochem. 2019;120(2):2532–9. 30206979 10.1002/jcb.27545

[14] Wu MS, Chien CC, Chang J, Chen YC. Pro-apoptotic effect of haem oxygenase‐1 in human colorectal carcinoma cells via endoplasmic reticular stress. J Cell Mol Med. 2019;23(8):5692–704. 31199053 10.1111/jcmm.14482 PMC6653387

[15] Doyle KM, Kennedy D, Gorman AM, Gupta S, Healy SJ, Samali A. Unfolded proteins and endoplasmic reticulum stress in neurodegenerative disorders. J Cell Mol Med. 2011;15(10):2025–39. 21722302 10.1111/j.1582-4934.2011.01374.x PMC4394214

[16] Cheng C, Dong W. Aloe-emodin induces endoplasmic reticulum stress-dependent apoptosis in colorectal cancer cells. Med Sci Monitor: Int Med J Experimental Clin Res. 2018;24:6331. 10.12659/MSM.908400 PMC6142869 30199885

[17] Nesran ZNM, Shafie NH, Ishak AH, Esa NM, Ismail A, Tohid SFM. Induction of endoplasmic reticulum stress pathway by green tea epigallocatechin-3-gallate (EGCG) in colorectal cancer cells: activation of PERK/p-eIF2α/ATF4 and IRE1α. BioMed Research International. 2019;2019. 10.1155/2019/3480569 PMC6942794 31930117

[18] Singh MP, Han J, Kang SC. 3′, 5-dihydroxy-3, 4′, 7-trimethoxyflavone-induces ER-stress-associated HCT-116 programmed cell death via redox signaling. Biomed Pharmacother. 2017;88:151–61. 28103509 10.1016/j.biopha.2017.01.027

[19] Roberts JL, Poklepovic A, Booth L. Curcumin interacts with sildenafil to kill GI tumor cells via endoplasmic reticulum stress and reactive oxygen/nitrogen species. Oncotarget. 2017;8(59):99451. 29245915 10.18632/oncotarget.19807 PMC5725106

[20] Zhang X, Sukamporn P, Zhang S, Min K-W, Baek SJ. 3, 3’-diindolylmethane downregulates cyclin D1 through triggering endoplasmic reticulum stress in colorectal cancer cells. Oncol Rep. 2017;38(1):569–74. 28586058 10.3892/or.2017.5693

[21] Wen Y, Zhang ZJ, Huang YP, Wang KP, Liu K, Zou H, et al. Application of the ethyl acetate extract of *cichorium* as a potential photosensitizer in photodynamic therapy induces apoptosis and autophagy in colorectal cancer cell lines via the protein kinase r-like Endoplasmic reticulum kinase pathway. J Biomed Nanotechnol. 2019;15(9):1867–80. 31387675 10.1166/jbn.2019.2825

[22] Kim JL, Kim BR, Kim DY, Jeong YA, Jeong S, Na YJ, et al. Cannabidiol enhances the therapeutic effects of TRAIL by upregulating DR5 in colorectal cancer. Cancers (Basel). 2019. 10.3390/cancers11050642. 31075907 10.3390/cancers11050642 PMC6562873

[23] Qiu Y, Li C, Zhang B, Gu Y. Bixin Prevents Colorectal Cancer Development through AMPK-Activated Endoplasmic Reticulum Stress. BioMed Research International. 2022;2022. 10.1155/2022/9329151 PMC8894005 35252457

[24] Lee DH, Sung KS, Guo ZS, Kwon WT, Bartlett DL, Oh SC, et al. TRAIL-induced caspase activation is a prerequisite for activation of the endoplasmic reticulum stress-induced signal transduction pathways. J Cell Biochem. 2016;117(5):1078–91. 26212606 10.1002/jcb.25289

[25] Zhang YS, Wang F, Cui SX, Qu XJ. Natural dietary compound naringin prevents azoxymethane/dextran sodium sulfate-induced chronic colorectal inflammation and carcinogenesis in mice. Cancer Biol Ther. 2018;19(8):735–44. 29580144 10.1080/15384047.2018.1453971 PMC6067845

[26] Hu YL, Yin Y, Liu HY, Feng YY, Bian ZH, Zhou LY, et al. Glucose deprivation induces chemoresistance in colorectal cancer cells by increasing ATF4 expression. World J Gastroenterol. 2016;22(27):6235–45. 27468213 10.3748/wjg.v22.i27.6235 PMC4945982

[27] Piao MJ, Han X, Kang KA, Fernando PDSM, Herath HMUL, Hyun JW. The endoplasmic reticulum stress response mediates shikonin-induced apoptosis of 5-fluorouracil–resistant colorectal cancer cells. Biomol Ther. 2022;30(3):265–73. 10.4062/biomolther.2021.118 PMC9047496 34607978

[28] Wang QY, Wang P, Xiao ZG. Resistant starch prevents tumorigenesis of dimethylhydrazine-induced colon tumors via regulation of an ER stress-mediated mitochondrial apoptosis pathway. Int J Mol Med. 2018;41(4):1887–98. 29393371 10.3892/ijmm.2018.3423 PMC5810243

[29] Chern YJ, Wong JCT, Cheng GSW, Yu A, Yin Y, Schaeffer DF, et al. The interaction between SPARC and GRP78 interferes with ER stress signaling and potentiates apoptosis via PERK/eIF2α and IRE1α/XBP-1 in colorectal cancer. Cell Death Dis. 2019. 10.1038/s41419-019-1687-x. 31243264 10.1038/s41419-019-1687-x PMC6594974

[30] Chiu CF, Lai GY, Chen CH, Chiu CC, Hung SW, Chang CF. 6,7-dihydroxy-2-(4′-hydroxyphenyl)naphthalene induces HCT116 cell apoptosis through activation of Endoplasmic reticulum stress and the extrinsic apoptotic pathway. Drug Des Devel Ther. 2019;13:1609–21. 31190740 10.2147/DDDT.S193914 PMC6512798

[31] Edagawa M, Kawauchi J, Hirata M, Goshima H, Inoue M, Okamoto T, et al. Role of activating transcription factor 3 (ATF3) in endoplasmic reticulum (ER) stress-induced sensitization of p53-deficient human colon cancer cells to tumor necrosis factor (TNF)-related apoptosis-inducing ligand (TRAIL)-mediated apoptosis through up-regulation of death receptor 5 (DR5) by Zerumbone and celecoxib. J Biol Chem. 2014;289(31):21544–61. 24939851 10.1074/jbc.M114.558890 PMC4118115

[32] Zhang X, Lee SH, Min KW, McEntee MF, Jeong JB, Li Q, et al. The involvement of endoplasmic reticulum stress in the suppression of colorectal tumorigenesis by tolfenamic acid. Cancer Prev Res. 2013;6(12):1337–47. 10.1158/1940-6207.CAPR-13-0220 PMC3855893 24104354

[33] Wu MS, Chien CC, Jargalsaikhan G, Ilsan NA, Chen YC. Activation of PERK contributes to apoptosis and G2/M arrest by microtubule disruptors in human colorectal carcinoma cells. Cancers. 2020;12(1). 10.3390/cancers12010097 PMC7017320 31906029

[34] Qi J, Zhou N, Li L, Mo S, Zhou Y, Deng Y, et al. Ciclopirox activates PERK-dependent endoplasmic reticulum stress to drive cell death in colorectal cancer. Cell Death Dis. 2020. 10.1038/s41419-020-02779-1. 32719342 10.1038/s41419-020-02779-1 PMC7385140

[35] Lu XS, Qiao YB, Li Y, Yang B, Chen MB, Xing CG. Preclinical study of cinobufagin as a promising anti-colorectal cancer agent. Oncotarget. 2017;8(1):988–98. 27894091 10.18632/oncotarget.13519 PMC5352212

[36] Stolfi C, Sarra M, Caruso R, Fantini MC, Fina D, Pellegrini R, et al. Inhibition of colon carcinogenesis by 2-methoxy-5-amino-N-hydroxybenzamide, a novel derivative of Mesalamine. Gastroenterology. 2010;138(1):221–30. 19737563 10.1053/j.gastro.2009.08.062

[37] Flocke LS, Trondl R, Jakupec MA, Keppler BK. Molecular mode of action of NKP-1339-a clinically investigated ruthenium-based drug - involves ER- and ROS-related effects in colon carcinoma cell lines. Invest New Drugs. 2016;34(3):261–8. 26988975 10.1007/s10637-016-0337-8 PMC4859864

[38] Wernitznig D, Kiakos K, Del Favero G, Harrer N, MacHat H, Osswald A, et al. First-in-class ruthenium anticancer drug (KP1339/IT-139) induces an immunogenic cell death signature in colorectal spheroids: in vitro. Metallomics. 2019;11(6):1044–8. 30942231 10.1039/c9mt00051h

[39] Skender B, Hofmanova J, Slavik J, Jelinkova I, Machala M, Moyer MP, et al. DHA-mediated enhancement of TRAIL-induced apoptosis in colon cancer cells is associated with engagement of mitochondria and specific alterations in sphingolipid metabolism. Biochimica et Biophysica Acta (BBA). 2014;1841(9):1308–17. 24953781 10.1016/j.bbalip.2014.06.005

[40] Lei Y, He L, Yan C, Wang Y, Lv G. PERK activation by CCT020312 chemosensitizes colorectal cancer through inducing apoptosis regulated by ER stress. Biochem Biophys Res Commun. 2021;557:316–22. 33894420 10.1016/j.bbrc.2021.03.041

[41] Shi Y, Wang X, Meng Y, Ma J, Zhang Q, Shao G, et al. A novel mechanism of endoplasmic reticulum stress- and c-Myc-degradation-mediated therapeutic benefits of antineurokinin-1 receptor drugs in colorectal cancer. Adv Sci. 2021. 10.1002/advs.202101936. 10.1002/advs.202101936 PMC8564433 34605226

[42] Rozpedek W, Pytel D, Dziki L, Nowak A, Dziki A, Diehl JA, et al. Inhibition of PERK-dependent pro-adaptive signaling pathway as a promising approach for cancer treatment. Polski Przeglad Chirurgiczny/ Pol J Surg. 2017;89(3):7–10. 28703114

[43] Rozpedek W, Pytel D, Wawrzynkiewicz A, Siwecka N, Dziki A, Dziki L, et al. Use of Small-molecule inhibitory compound of PERK-dependent signaling pathway as a promising Target-based therapy for colorectal cancer. Curr Cancer Drug Targets. 2020;20(3):223–38. 31906838 10.2174/1568009620666200106114826

[44] Kong FH, Zou H, Liu X, He J, Zheng YW, Xiong L, et al. MiR-7112-3p targets PERK to regulate the Endoplasmic reticulum stress pathway and apoptosis induced by photodynamic therapy in colorectal cancer CX-1 cells. Photodiagn Photodyn Ther. 2020. 10.1016/j.pdpdt.2020.101663. 10.1016/j.pdpdt.2020.101663 31945549

[45] Xu K, Han B, Bai Y, Ma XY, Ji ZN, Xiong Y, et al. MiR-451a suppressing BAP31 can inhibit proliferation and increase apoptosis through inducing ER stress in colorectal cancer. Cell Death Dis. 2019. 10.1038/s41419-019-1403-x. 30770794 10.1038/s41419-019-1403-x PMC6377610

[46] De Simone V, Bevivino G, Sedda S, Izzo R, Laudisi F, Dinallo V et al. Smad7 knockdown activates protein kinase RNA-associated eIF2 alpha pathway leading to colon cancer cell death. Cell Death Dis. 2017;8. 10.1038/cddis.2017.103 PMC5386514 28300830

[47] Spaan CN, Smit WL, de Jeude JFV, Meijer BJ, Muncan V, van den Brink GR et al. Expression of UPR effector proteins ATF6 and XBP1 reduce colorectal cancer cell proliferation and sternness by activating PERK signaling. Cell Death Dis. 2019;10. 10.1038/s41419-019-1729-4 PMC6588629 31227689

[48] Cui C, Ye L, Huang Z, Huang S, Liu H, Yu J. FAM172A is a tumor suppressor in colorectal carcinoma. Tumor Biol. 2016;37(5):6501–10. 10.1007/s13277-015-4166-8 26637224

[49] Kumara M, Piao MJ, Kang KA, Ryu YS, Park JE, Shilnikova K, et al. Non-thermal gas plasma-induced Endoplasmic reticulum stress mediates apoptosis in human colon cancer cells. Oncol Rep. 2016;36(4):2268–74. 27573888 10.3892/or.2016.5038

[50] Rossi A, Pakhomova ON, Mollica PA, Casciola M, Mangalanathan U, Pakhomov AG, et al. Nanosecond pulsed electric fields induce endoplasmic reticulum stress accompanied by immunogenic cell death in murine models of lymphoma and colorectal cancer. Cancers (Basel). 2019. 10.3390/cancers11122034. 31861079 10.3390/cancers11122034 PMC6966635

[51] Lai MC, Chang CM, Sun HS. Hypoxia induces autophagy through translational up-regulation of lysosomal proteins in human colon cancer cells. PLoS One. 2016. 10.1371/journal.pone.0153627. 27078027 10.1371/journal.pone.0153627 PMC4831676

[52] Zheng H, Wu Y, Guo T, Liu F, Xu Y, Cai S. Hypoxia Induces Growth Differentiation Factor 15 to Promote the Metastasis of Colorectal Cancer via PERK-eIF2 α Signaling. BioMed Research International. 2020;2020. 10.1155/2020/5958272 PMC7008299 32076610

[53] Cernigliaro C, D’Anneo A, Carlisi D, Giuliano M, Gammazza AM, Barone R, et al. Ethanol-mediated stress promotes autophagic survival and aggressiveness of colon cancer cells via activation of Nrf2/HO-1 pathway. Cancers (Basel). 2019. 10.3390/cancers11040505. 30974805 10.3390/cancers11040505 PMC6521343

[54] Zhang YH, Cui SX, Wan SB, Wu SH, Qu XJ. Increased S1P induces S1PR2 internalization to blunt the sensitivity of colorectal cancer to 5-fluorouracil via promoting intracellular uracil generation. Acta Pharmacol Sin. 2020. 10.1038/s41401-020-0460-0. 32647340 10.1038/s41401-020-0460-0 PMC8027438

[55] Jiang X, Li D, Wang G, Liu J, Su X, Yu W, et al. Thapsigargin promotes colorectal cancer cell migration through upregulation of LncRNA MALAT1. Oncol Rep. 2020;43(4):1245–55. 32323831 10.3892/or.2020.7502 PMC7057937

[56] Colangelo T, Polcaro G, Ziccardi P, Muccillo L, Galgani M, Pucci B, et al. The miR-27a-calreticulin axis affects drug-induced immunogenic cell death in human colorectal cancer cells. Cell Death Dis. 2016;7(2):e2108. 26913599 10.1038/cddis.2016.29 PMC4849155

[57] Liang G, Fang X, Yang Y, Song Y. Knockdown of CEMIP suppresses proliferation and induces apoptosis in colorectal cancer cells: downregulation of GRP78 and attenuation of unfolded protein response. Biochem Cell Biol. 2018;96(3):332–41. 29024602 10.1139/bcb-2017-0151

[58] Matassa DS, Amoroso MR, Agliarulo I, Maddalena F, Sisinni L, Paladino S, et al. Translational control in the stress adaptive response of cancer cells: A novel role for the heat shock protein TRAP1. Cell Death Dis. 2013. 10.1038/cddis.2013.379. 24113185 10.1038/cddis.2013.379 PMC3824688

[59] Bray F, Ferlay J, Soerjomataram I, Siegel RL, Torre LA, Jemal A. Global cancer statistics 2018: GLOBOCAN estimates of incidence and mortality worldwide for 36 cancers in 185 countries. Cancer J Clin. 2018;68(6):394–424. 10.3322/caac.21492 30207593

[60] Luo B, Lee AS. The critical roles of endoplasmic reticulum chaperones and unfolded protein response in tumorigenesis and anticancer therapies. Oncogene. 2013;32(7):805–18. 22508478 10.1038/onc.2012.130 PMC3819728

[61] Alnuqaydan AM, Rah B, Almutary AG, Chauhan SS. Synergistic antitumor effect of 5-fluorouracil and withaferin-A induces endoplasmic reticulum stress-mediated autophagy and apoptosis in colorectal cancer cells. Am J Cancer Res. 2020;10(3):799–815. 32266092 PMC7136917

[62] Cheng C, Dong W. Aloe-emodin induces endoplasmic reticulum stress-dependent apoptosis in colorectal cancer cells. Med Sci Monit. 2018;24:6331–9. 30199885 10.12659/MSM.908400 PMC6142869

[63] Roberts JL, Poklepovic A, Booth L. Curcumin interacts with sildenafil to kill GI tumor cells via Endoplasmic reticulum stress and reactive oxygen/nitrogen species. Oncotarget. 2017;8(59):99451–69. 29245915 10.18632/oncotarget.19807 PMC5725106

[64] Wu MS, Chien CC, Chang J, Chen YC. Pro-apoptotic effect of haem oxygenase-1 in human colorectal carcinoma cells via endoplasmic reticular stress. J Cell Mol Med. 2019;23(8):5692–704. 31199053 10.1111/jcmm.14482 PMC6653387

[65] Singh MP, Han J, Kang SC. 3 ‘,5-dihydroxy-3,4 ‘,7-trimethoxyflavone-induces ER-stress-associated HCT-116 programmed cell death via redox signaling. Biomed Pharmacother. 2017;88:151–61. 28103509 10.1016/j.biopha.2017.01.027

