# Supplementary material for: Importance of PERK pathway modulation on colorectal cancer management: a systematic review
Source: BMC Cancer. 2025 Oct 3;25:1502. doi: 10.1186/s12885-025-14952-w (PMC12495646; doi:10.1186/s12885-025-14952-w)
Supplement: Supplementary file 1 — Supplementary material 1. [file 12885_2025_14952_MOESM1_ESM.docx]

**Search Strategy**

The following search string was used across all databases: **(colorectal cancer[Title/Abstract]) AND (PERK[Title/Abstract])**

**Databases Searched**

- **PubMed**
- **Scopus**
- **Web of Science**

**Search Parameters**

- **Date of Search:** [Insert exact date or month/year]
- **Date Range:** No restrictions applied; all available records up to the search date were included
- **Language Restriction:** English only
- **Publication Type Restriction:** None applied during initial search

**Search Results**

| **Database** | **Records Retrieved** |
| --- | --- |
| PubMed | 139 |
| Scopus | 111 |
| Web of Science | 133 |
| **Total** | **383** |

**Deduplication**

- Duplicate records removed: 169
- Remaining for screening: 214

**Screening Outcomes**

| **Screening Category** | **Number of Articles** |
| --- | --- |
| Review articles | 8 |
| Unrelated articles | 162 |
| **Relevant articles** | **44** |

**Notes**

- Screening was performed manually based on title and abstract relevance to PERK and colorectal cancer.
- Full-text review was conducted for the 44 included studies.
