# Supplementary figures and images for: Importance of PERK pathway modulation on colorectal cancer management: a systematic review
Source: BMC Cancer. 2025 Oct 3;25:1502. doi: 10.1186/s12885-025-14952-w (PMC12495646; doi:10.1186/s12885-025-14952-w)

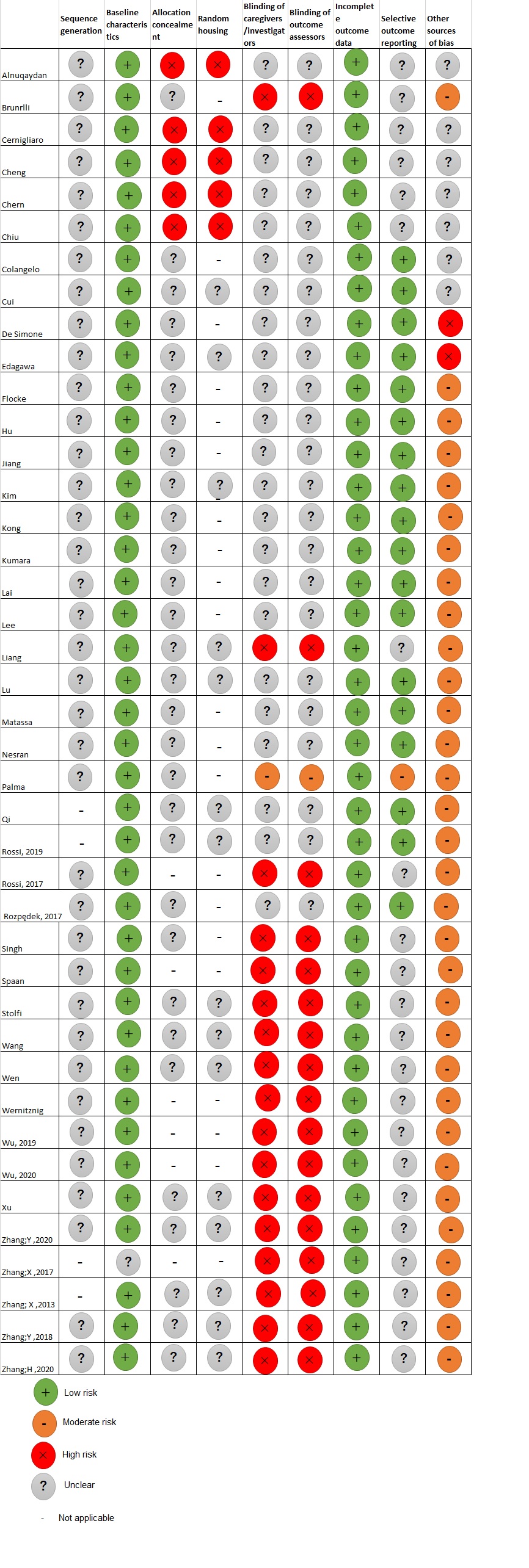

Supplement: Supplementary file 2 — Supplementary material 2. [file 12885_2025_14952_MOESM2_ESM.jpg]
